# Development and External Validation of Machine Learning-Based Models for Predicting Lung Metastasis in Kidney Cancer: A Large Population-Based Study

**DOI:** 10.1155/2023/8001899

**Published:** 2023-06-20

**Authors:** Xinglin Yi, Yuhan Zhang, Juan Cai, Yu Hu, Kai Wen, Pan Xie, Na Yin, Xiangdong Zhou, Hu Luo

**Affiliations:** ^1^Department of Respiratory and Critical Care Medicine, The First Affiliated Hospital of the Army Medical University, Chongqing, China; ^2^Department of Renal Dialysis Center, The First Affiliated Hospital of the Army Medical University, Chongqing, China

## Abstract

The accuracy of indices widely used to evaluate lung metastasis (LM) in patients with kidney cancer (KC) is insufficient. Therefore, we aimed at developing a model to estimate the risk of developing LM in KC based on a large population size and machine learning algorithms. Demographic and clinicopathologic variables of patients with KC diagnosed between 2004 and 2017 were retrospectively analyzed. We performed a univariate logistic regression analysis to identify risk factors for LM in patients with KC. Six machine learning (ML) classifiers were established and tuned using the ten-fold cross-validation method. External validation was performed using clinicopathologic information from 492 patients from the Southwest Hospital, Chongqing, China. Algorithm performance was estimated by analyzing the area under the receiver operating characteristic curve (AUC), accuracy, sensitivity, specificity, precision, recall, F1 score, clinical decision analysis (DCA), and clinical utility curve (CUC). A total of 52,714 eligible patients diagnosed with KC were enrolled, of whom 2,618 developed LM. Variables of age, sex, race, T stage, N stage, tumor size, histology, and grade were identified as important for the prediction of LM. The extreme gradient boosting (XGB) algorithm performed better than other models in both the internal validation (AUC: 0.913, sensitivity: 0.873, specificity: 0.809, and F1 score: 0.325) and the external validation (AUC: 0.904, sensitivity: 0.750, specificity: 0.878, and F1 score: 0.364). This study established a predictive model for LM in KC patients based on ML algorithms which showed high accuracy and applicative value. A web-based predictor was built using the XGB model to help clinicians make more rational and personalized decisions.

## 1. Introduction

Kidney cancer (KC) originates in the kidney and accounts for approximately 2% of all malignancies worldwide [1, 2]. Approximately 350,000 people are newly diagnosed with KC, and 15,000 die from this cancer yearly [3]. According to the 2016 World Health Organization classification of urinary carcinoma, KC incorporates several subtypes, including renal cell carcinoma (90%), transitional cell carcinoma (1%), renal sarcoma (1%), and other kidney tumors [4]. Most patients have a favorable prognosis, and more than half of the patients have an overall survival (OS) of more than ten years.

Although immunotherapy and precision surgery provide patients with KC with a more favorable prognosis, approximately 20% of them have distant metastasis at the time of diagnosis. Once the cancer has spread, the 5-year OS rate severely decreases to approximately 10% [5]. The lungs are the most common site of distant metastasis, accounting for 55% of all metastatic cases of KC [6]. Previous studies demonstrated that although drug treatment is advanced, patients who develop lung metastasis (LM) only have a median survival time of 15 months [7]. Therefore, precise measures to diagnose LM will provide clinicians with more rational decisions. Conventional contrast-enhanced computed tomography (CT) is traditionally used for preoperative diagnosis. However, CT has relatively low sensitivity (62%) and specificity (86%) in predicting LM from KC, leading to the misdiagnosis of many patients with LM resulting in unnecessary surgeries that cannot cure their cancer [8]. Moreover, because the metastatic focus is usually small, many patients do not exhibit respiratory symptoms, resulting in a delayed diagnosis of LM. Although magnetic resonance imaging and biopsy offered high accuracy in detecting LM, the high financial cost and long waiting duration delayed the diagnosis of LM and thus limited its application to all patients with KC [9]. Therefore, a predictive model that conveniently and precisely detects LM in patients with KC is needed, which could help clinicians make more rational treatment decisions, adopt preventive therapy, and improve patient survival. The tumor-node-metastasis (TNM) staging system, UCLA Integrated Staging System (UISS), the tumor stage, size, grade, necrosis (SSIGN) score, and Leibovich score encompass common pathological factors and are frequently used to assess the recurrence and metastatic risk of KC in clinical studies. However, the C-index of these predictive systems was reported to range between 0.723 and 0.80 [10, 11] and was not highly satisfactory.

Machine learning (ML) has emerged as a powerful tool in various fields such as computer vision, security systems, and medicine, where it has shown significant value [12, 13]. An increasing number of studies are demonstrating its potential to improve diagnostics, prognostic predictions, and treatment planning across a range of clinical diseases. In the medicine field, ML algorithms can learn from and make predictions based on data, enabling the creation of personalized, data-driven models that can enhance clinical decision-making and patient care [14]. For instance, Liu et al. employed machine learning models based on a population of 311,408 to predict bone metastasis in patients with ductal carcinoma. They achieved an area under the receiver operating characteristic curve (AUC) of 0.888, a sensitivity of 0.801, and a specificity of 0.837. This high predictive accuracy highlights the potential for ML models to be utilized in determining appropriate treatment strategies for such patients [15]. Similarly, Cheng et al. developed a machine learning-based model using a population of 10,580 to predict the survival of patients with neuroendocrine tumors. They attained an AUC of 0.90, which was significantly greater than that of the American Joint Committee on Cancer (AJCC) seventh staging system. The success of this model demonstrates the utility of ML-based approaches for prognostication and guiding clinical decision-making in oncology [16]. In this study, we attempted to build an exact tool based on ML algorithms by employing a large population of patients with KC from the Surveillance, Epidemiology, and End Results (SEER) database and a real-world hospital.

## 2. Materials and Methods

### 2.1. Patients

The patients were extracted from the SEER database (2010–2017), which comprises approximately 30% of the total population in the USA [17]. Patients from the Southwest Hospital in China were also enrolled in the patient cohort. The inclusion criteria were patients with kidney malignancy. Patients who (1) were younger than 18 years old, (2) had unknown T or N stage, (3) had unknown LM, (4) had unknown tumor size, (5) had more than one primary tumor site, and (6) had unknown tumor grade were excluded from the cohort. This retrospective study involving human participants was conducted in accordance with the ethical standards of the institutional and national research committee. Ethical approval was waived by the local Ethics Committee of Southwest Hospital in view of the retrospective nature of the study.

**Figure 1:**
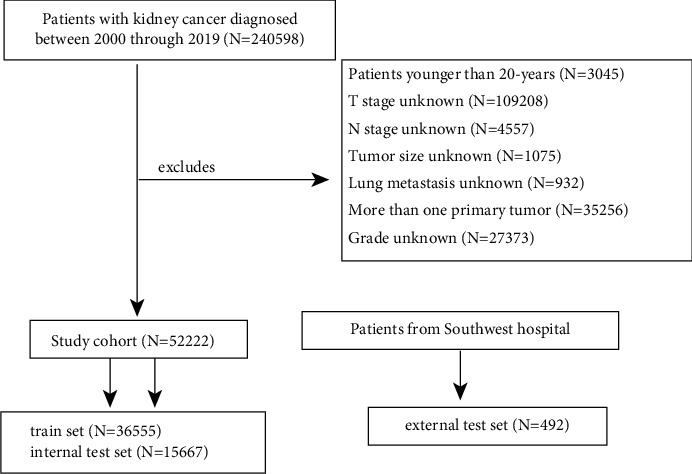
Detailed flowchart for the patient selection procedure.

Finally, 52,714 patients were enrolled in this study. We randomly assigned 52,222 patients from the SEER database to the training (70%) and internal test (30%) sets. The training set was used to establish the predictive models, while the latter was used to validate the model's performance. Subsequently, 492 patients from Southwest Hospital were assigned to the external test cohort, which was used to externally re-validate the models. A detailed patient selection flowchart is shown in Figure 1.

### 2.2. Feature Selection

We retrospectively selected clinical features using SEERStat software (8.4.0.1) to screen for commonly used variables, including age, sex, T stage, N stage, laterality, tumor size, grade, histology, race, and LM. The T and N stages were determined according to the seventh edition of the AJCC TNM staging system. Histology categories included the following: (8120) transitional cell carcinoma, (8255) adenocarcinoma with mixed subtypes, (8260) papillary adenocarcinoma, (8310) clear cell adenocarcinoma, (8312) renal cell carcinoma, and (8317) chromophobe renal carcinoma and other rare subtypes.

### 2.3. Model Establishment and Model Performance

First, we used univariate logistic regression to identify features related to LM in the training cohort and then included variables with a *P* value less than 0.05 in the model development process. We measured feature importance using the permutation method in machine learning models, as described in references [14, 18]. Then, utilizing the “Tidymodels” packages, we constructed six machine learning models that incorporated the selected features. These models included logistic regression (LR), extreme gradient boosting (XGB), random forest (RF), support vector machine (SVM), artificial neural network (ANN), and decision tree (DT). These models were developed using the selected variables from the procedures described above and applied to the training cohort. The hyperparameters were optimized using a ten-fold cross-validation and grid search approach, with the specific parameter settings detailed in Supplementary file 1.

Several evaluators, including AUC, accuracy, sensitivity, specificity, precision, recall, and F1 score, were used to estimate the performance of models in internal and external test cohorts. Decision curve analysis (DCA) and clinical utility curve (CUC) were performed to examine the discriminative and fitting abilities of the models. We then selected the best-performing model to build a web-based online calculator for generalization. In addition, to evaluate the contribution of each variable in prediction, we used an imputation-based method to rank the importance of the selected parameters in the training cohort. Finally, the survival analysis of OS and cancer-specific survival was performed to validate the prognostic value using the Kaplan–Meier method based on the predictive results.

### 2.4. Statistical Analysis

The age and tumor size variables in this study were measured in a continuous form, and the *t*-test was used to compare the differences between these two variables. The TNM stage was classified according to the 7th AJCC TNM classification. Other variables were displayed in the categorical form, and the chi-square test was used to compare the differences. A correlation analysis by the Spearman method was performed to describe the relevance among variables and identify highly relevant features to LM. The relevant index categorized three levels: 0–0.4, low; 0.4–0.7, intermediate; and ≥0.7, high. All statistical analyses were performed using R software (version 4.2.1; R Foundation for Statistical Computing).

## 3. Results

### 3.1. Baseline Characteristics

In total, 52,714 patients were enrolled in this study. Among them, 2,618 (4.96%) patients with KC were diagnosed with LM. A comparison of characteristics between the LM and non-LM cohorts is summarized in Table 1. Compared with non-LM individuals, individuals with LM were more likely to be elderly (61.3 vs. 59.6), male (71.4% vs. 62.5%), with larger tumor sizes (51.0 mm vs. 34.7 mm), advanced (T3, T4) T stage (71.8% vs. 20.1%) and N stage (33% vs. 2.9%), and higher (III-IV) tumor grade (79.2% vs. 28.4%).

After being randomly divided into training and internal test groups in a 7 : 3 ratio, patients in the training arm (38,335) had characteristics similar to those of the internal (15,667) and external (492) arms (Table 2).

### 3.2. Univariate and Multivariate Logistic Regression

Based on univariate regression analysis, variables of age, sex, T stage, N stage, tumor size, histology, race, and tumor grade were features with a *P* < 0.05 (Table 3). These variables were used in building six ML algorithms. Multivariate regression analysis showed that older age, male sex, larger tumor size, Asian ethnicity, advanced T and N stage, tumor grade, and histology of renal cell carcinoma were identified as independent factors for LM.

### 3.3. Correlation Analysis

**Figure 2:**
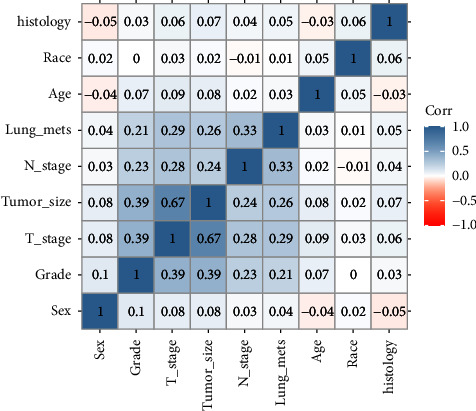
Correlation analysis based on Spearman analysis. The brighter the colors, the higher the relevance among variables. In the center of each small square is displayed the index of correlation. Lung_mets: lung metastasis.

To recognize the variables relevant to LM and to examine the linear relationship among characteristics, we performed a correlation analysis based on the Spearman method. As shown in Figure 2, no variables exhibited a high linear relationship (index >0.8). In addition, the Spearman relevant analysis showed that characteristics of N stage, T stage, tumor size, and tumor grade were LM's four most relevant features.

### 3.4. Model Performance

**Figure 3:**
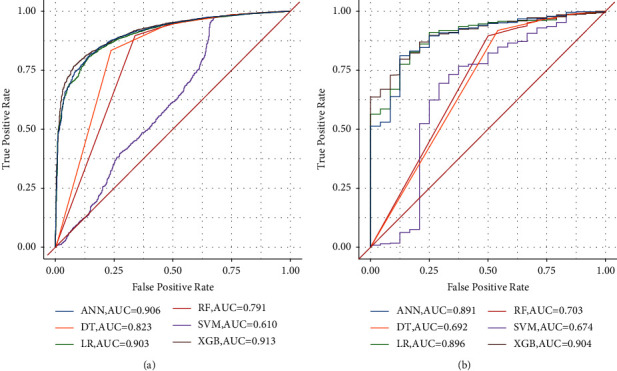
Receiver operating characteristic curve of six algorithms in the internal (a) and external test cohorts (b).

Receiver operating characteristic (ROC) curves of the internal and external cohorts are shown in Figure 3, indicating that the XGB algorithm exhibited the highest AUC value. Detailed information on the performance is shown in Table 4. In internal and external test cohorts, XGB outperformed the others, with AUC, accuracy, sensitivity, and specificity of 0.913, 0.812, 0.873, and 0.809, respectively, in the internal test and 0.904, 0.872, 0.750, and 0.878, respectively, in the external test. The XGB algorithm demonstrated the third highest F1 scores, following RF and SVM, in the internal test set, while it performed the best in the external test set. Overall, the XGB algorithm outperformed the others in terms of performance.

**Figure 4:**
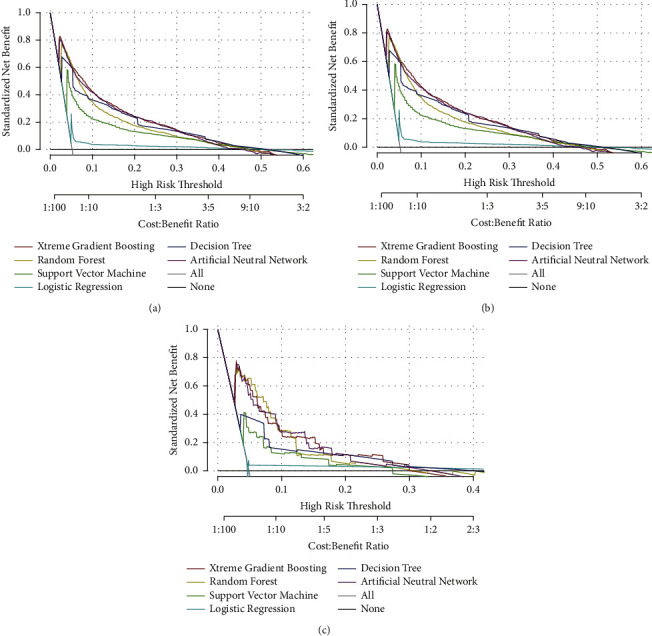
Decision curve analysis curves of algorithms in the training set (a), internal test set (b), and external test set (c).

**Figure 5:**
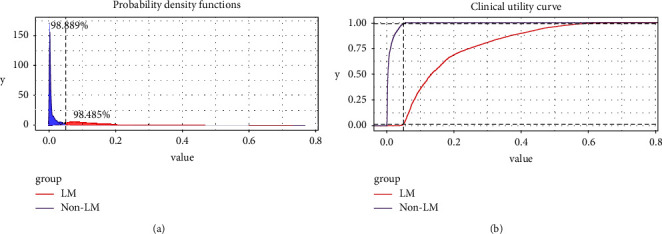
Probability density plot (a) and the clinical utility curve (b) of the extreme gradient boosting model.

As indicated in Figure 4, DCA curves suggested that XGB had the highest clinical applicability, which meant that clinicians would make a more accurate judgment using the XGB model rather than other ML algorithms. The probability density plot showed that the predictive probability distribution in non-LM patients was extremely high; whereas it was relatively flat in LM patients (Figure 5). A CUC was used to detect the optimal threshold of each predictive cohort. As shown in Figure 5, when the value of the *x*-axis was >0.05, the XGB model could accurately predict patients with LM.

### 3.5. Feature Importance Evaluation

**Figure 6:**
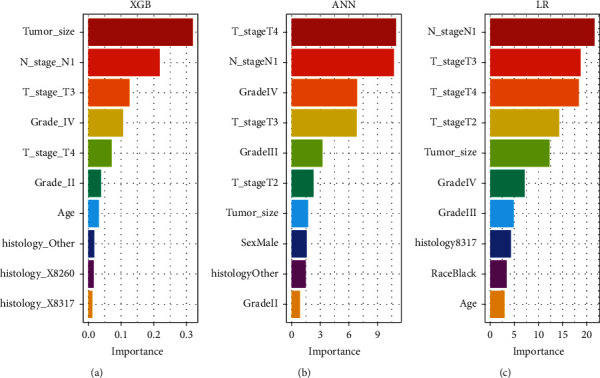
Feature importance rankings in extreme gradient boosting (a), artificial neural network (b), and logistic regression (c).

Based on the permutation test, we ranked variable importance for prediction in the three best-performing models (ANN, XGB, and LR). It is not difficult to observe that although the three ranks differ slightly, T stage, N stage, tumor size, and grade still ranked in the top five (Figure 6).

### 3.6. Calculator Online Establishment

**Figure 7:**
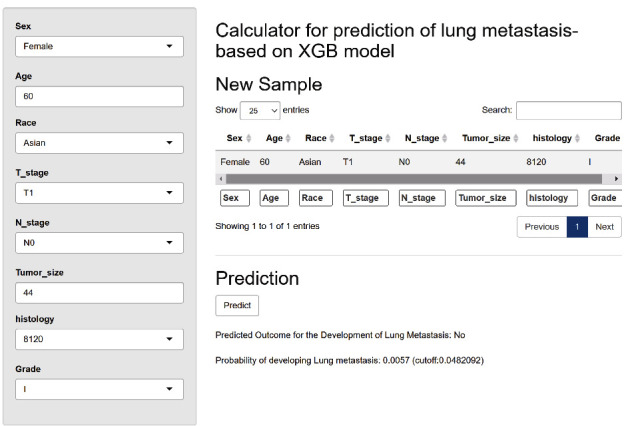
Web-based calculator using the extreme gradient boosting algorithm. The website was published online with the URL https://medicalmachinelearning.shinyapps.io/ModelForLungMetastasis/.

To generalize the predictive model based on the XGB, which performed best among the six algorithms, we built a web-based online predictor, which is available at https://medicalmachinelearning.shinyapps.io/ModelForLungMetastasis/. As shown in Figure 7, as long as the accessible variables are entered into the option box, we can predict the risk of LM in KC. For example, if we select “female” for gender, “60” for age, “Asian” for race, “T1-N0” for stage, “44 millimeters” for tumor size, “8120 (transitional cell carcinoma)” for histology, and “grade I” in the calculator's input fields, and then press the “Predict” button, the predicted outcome for developing LM will be “No.” This indicates that, based on these inputs, it is less likely for the patient to develop LM.

### 3.7. Survival Analysis

**Figure 8:**
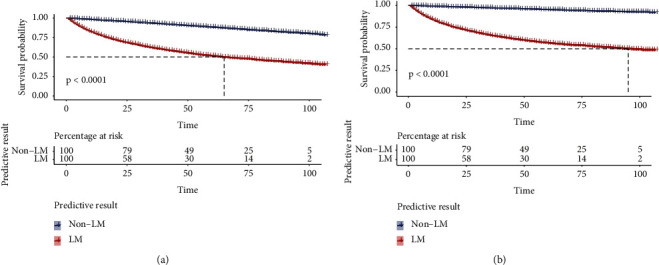
Survival analysis of overall survival (a) and cancer-specific survival (b) based on the extreme gradient boosting algorithm using the Kaplan–Meier method. Log-rank analysis was used to determine the *P* value.

Based on the predictive results of the XGB model, we performed a survival analysis using the Kaplan–Meier method. As suggested in Figure 8, those who were determined to have LM had a significantly shorter survival time (*P* < 0.0001) than those who did not, suggesting a good discriminative ability of the XGB model. Thus, the XGB model can also help clinicians judge the prognosis of patients with KC.

## 4. Discussion

KC is a prevalent urinary cancer with a relatively long survival time in patients without distant metastasis. However, the prognosis was severely impaired in the case of distant metastases, and the 5-year survival rate of those patients was only 12% [19, 20]. As reported in prior studies, the lung was the most common site for distant metastasis, covering approximately 45–50% of all metastatic cases with a poor prognosis of only 7 months [6, 21–23]. Regarding treatment, due to the high resistance to chemoradiotherapy exhibited by this disease, surgical resection was still deemed the most effective treatment for curing KC. However, many patients who underwent surgical treatment were still at risk for LM. Recently, Choueiri et al. demonstrated that adjuvant pembrolizumab after surgical resection significantly prolonged the disease-free survival to 24 months, but adverse events were common (with an incidence of 21.3%) and reduced the OS owing to these adverse effects [24].

In the era of targeted therapy, numerous targeted treatments have been successfully developed, leading to improved clinical outcomes for patients with KC. Multitargeted, small-molecule tyrosine kinase inhibitor (TKI) drugs that act against vascular endothelial growth factor receptors, platelet-derived growth factor receptors, and other kinases are recommended for patients with previously untreated advanced KC. These therapies have contributed to a significant improvement in median progression-free survival (PFS) from 5.5 to 11 months, as well as an increase in median OS from 23 to 26 months [25]. In recent years, immune checkpoint inhibitors (ICIs) have emerged as a crucial therapeutic approach. A meta-analysis has revealed that combining TKI drugs with immunotherapies significantly enhances tumor responses and improves survival outcomes for patients with metastatic KC. This finding suggests a promising future for the treatment of advanced KC [26]. With a personalized therapeutic schedule, the survival of patients with KC may be improved because it could prevent unsuitable patients from adjuvant treatment's adverse effects. Hence, early attention to those at risk for LM and taking personal preventative measures are important. To the best of our knowledge, risk factors for LM have been examined in several studies [27, 28]. However, there are only a few established predictive models. Lu et al. [20] used the SEER database, in which 10,929 patients were eligible for a nomogram construction, to predict the LM of renal cell carcinoma. Their study demonstrated that clear cell carcinoma pathology was a risk factor for LM compared to other subtypes of KC, which is consistent with our results. They also suggested that parameters such as race, grade, T stage, N stage, surgery, tumor size, and distant metastasis in other sites were independent variables for LM. Similarly, Xu et al. [29] developed machine learning-based models to evaluate the risk of developing lung metastasis in kidney cancer patients using the SEER database. They performed multivariate logistic regression and found that grade, T and N stage, tumor size, and metastasis to other sites, including the bone, brain, and liver, were all risk factors. Ultimately, they established a prediction model with a high AUC. However, Chan's study also had some limitations. For instance, their study was only conducted using the SEER database, and the model was validated using 10-fold cross-validation without being split into an internal test set. Furthermore, it did not undergo external validation, both of which may limit the generalizability of the model. Besides, these prediction models irrationally included variables of other metastatic sites, which is unsuitable for preoperative evaluation and would dramatically reduce the model's utility. Molecularly, certain circRNAs such as circ-EGLN3 and SCARB1 [30, 31] were demonstrated to promote effectiveness and predictive value for LM, but these molecular entities are difficult to examine in each person and include a high cost, thus greatly hampering their clinical application value.

As a technological tool, ML has yielded remarkable results in assisting epidemiologists [32]. Using ML algorithms, Handelman predicted a reduction in diagnostic errors by addressing complex and tedious clinical work [14]. Compared with conventional CT images frequently used in preoperative screening, ML models were good at assigning risk levels for developing LM with high accuracy and convenience. Here, we successfully developed a web-based predictor to predict the risk for LM in patients newly diagnosed with KC, which used easily accessible clinical characteristics and proved to be highly accurate and applicable.

This study used the data of 36,555 patients with KC to establish the ML models. These algorithms have been internally validated in 15,667 patients from the SEER cohort and externally tested in 492 patients from a Chinese cohort. Among the six predictive models, XGB performed the best, with an AUC of 0.913 and 0.904 in the internal and external test cohorts, respectively. DCA and CUC curves showed great discriminative and applicative abilities in the clinic.

Referring to LM risk factors, Thompson and colleagues suggested that a larger tumor size and an advanced T stage were significantly associated with a higher probability of metastasis in renal cell carcinoma [33]. Among 781 KC patients with tumors less than 3 cm, they identified only one patient with a record of distant metastasis. For every 1 cm increase in tumor size, the hazard ratio of metastasis-free survival increased by 0.24, with *P* < 0.001. Our study also yielded consistent results, indicating that larger tumor size was more strongly associated with LM and identified as an independent factor for LM. Mikami et al. reported that KC with higher grades tended to develop epithelial-mesenchymal transition (EMT), which has been proven to be critical for metastasis [34]. In our study, we observed a higher proportion of high-grade (III-IV) cases in the LM cohort and considered it an independent factor for LM. We also found that positive lymph nodes were more likely to be observed in the metastatic cohort and were one of the most influential characteristics associated with LM in KC patients. Similarly, Dudani et al. discovered that lymph node involvement contributed to distant metastasis and was common in KC, especially in papillary renal cell carcinoma [27]. Blacks, Asians, and African Americans have been reported to have a relatively high mortality risk due to KC [35]. This result may be resulted by higher probability of LM which was identified as a risk factor in this study. Consistently, Vaishampayan et al. found that compared with white patients with KC, black patients had a significantly shorter survival time (*P* < 0.0001) [36]. Histology also seemed to suggest distant metastasis of KC. Wang analyzed 36,365 patients with renal cell carcinoma and found that the clear cell subtype had a higher risk of distant metastasis, followed by the papillary and chromophobe subtypes [37]. Rong et al. reported that in multivariate analysis, compared with clear cell carcinoma, sarcomatoid had a relatively high hazard ratio for generating LM. Chromophobe cell carcinoma and collecting duct carcinoma are less likely to develop LM in KC [38]. In this study, we found that the clear cell carcinoma subtype was a risk estimator of LM, while chromophobe and papillary cell carcinomas had a relatively low incidence of LM, similar to previous studies. Moreover, multivariate LR showed that male patients had a tendency to develop LM, which may be correlated with a higher smoking rate in males and requires further investigation.

ML as a black box has a long-term problem of interpretability [39]. To address this problem, we built a web-based free calculator based on the XGB algorithm trained in this study to help clinicians rapidly predict and estimate the probability of LM in patients with KC.

Although the predictive model performed well in estimating LM in patients with KC, this study has several limitations. First, this was a retrospective study, inevitably resulting in a selection bias. Second, the study did not involve common clinical indices, such as marriage and biochemical indices [40]. Finally, the external validation cohort from China only involved Chinese individuals, so more validation arms in other countries are needed to examine the model's utility further.

## 5. Conclusions

In this study, we used a mainstream powerful machine learning tool to identify the high-risk factors of renal cancer lung metastasis and established a convenient and efficient web tool to help clinical doctors quickly identify those renal cancer patients prone to lung metastasis. This tool will greatly help patients in economically underdeveloped areas or those who are not convenient for puncture biopsy. The power behind this study is based on the large population in the SEER database, and the model has been independently verified by an external team. Future work should focus on increasing the sample size and diversity of ethnicities to validate the machine learning model. Additionally, incorporating more parameters, such as patient symptoms, may help improve performance. These are areas that could be addressed in future research.

## Figures and Tables

**Table 1:** Clinicopathologic characteristics.

Variables	Overall (*N* = 52,714)	Non-LM (*N* = 50,096)	LM (*N* = 2,618)
*Age*			
Mean (SD)	59.7 (12.3)	59.6 (12.4)	61.3 (11.0)

*Sex*			
Female	19,555 (37.1%)	18,805 (37.5%)	750 (28.6%)
Male	33,159 (62.9%)	31,291 (62.5%)	1,868 (71.4%)

*Laterality*			
Left	25,973 (48.8%)	24,395 (48.7%)	1,330 (50.3%)
Right	26,741 (50.3%)	25,233 (50.4%)	1,288 (48.7%)

*Race*			
Asian	3,625 (6.9%)	3,408 (6.8%)	217 (8.3%)
Black	5,701 (10.8%)	5,523 (11.0%)	178 (6.8%)
Others	1,031 (2.0%)	992 (2.0%)	39 (1.5%)
White	42,357 (80.4%)	40,173 (80.2%)	2,184 (83.4%)

*T_stage*			
T1	35,124 (66.6%)	34,842 (69.6%)	282 (10.8%)
T2	5,646 (10.7%)	5,189 (10.4%)	457 (17.5%)
T3	10,959 (20.8%)	9,465 (18.9%)	1,494 (57.1%)
T4	985 (1.9%)	600 (1.2%)	385 (14.7%)

*N_stage*			
N0	50,392 (95.6%)	48,646 (97.1%)	1,746 (66.7%)
N1	2,322 (4.4%)	1,450 (2.9%)	872 (33.3%)

*Tumor_size*			
Mean (SD)	53.6 (37.3)	51.1 (34.7)	101 (51.0)

*Histology*			
8120	182 (0.3%)	147 (0.3%)	35 (1.3%)
8255	1,344 (2.5%)	1,219 (2.4%)	125 (4.8%)
8260	6,029 (11.4%)	5,931 (11.8%)	98 (3.7%)
8310	35,878 (68.1%)	34,176 (68.2%)	1,702 (65.0%)
8312	5,736 (10.9%)	5,351 (10.7%)	385 (14.7%)
8317	2,210 (4.2%)	2,190 (4.4%)	20 (0.8%)
Others	1,335 (2.5%)	1,082 (2.2%)	253 (9.7%)

*Grade*			
I	5,923 (11.2%)	5,862 (11.7%)	61 (2.3%)
II	26,761 (50.8%)	26,280 (52.5%)	481 (18.4%)
III	15,484 (29.4%)	14,401 (28.7%)	1,083 (41.4%)
IV	4,546 (8.6%)	3,553 (7.1%)	993 (37.9%)

LM, lung metastasis; 8120, transitional cell carcinoma; 8255, adenocarcinoma with mixed subtypes; 8260, papillary adenocarcinoma; 8310, clear cell adenocarcinoma; 8312, renal cell carcinoma; 8317, chromophobe renal carcinoma.

**Table 2:** Characteristics in the training, internal, and external test cohorts.

Variables	Training cohort		Internal test cohort		External test cohort	
	Non-LM	LM	Non-LM	LM	Non-LM	LM
Total	*N* = 34,775	*N* = 1,780	*N* = 14,853	*N* = 814	*N* = 468	*N* = 24
*Age*						
Mean (SD)	59.6 (12.4)	61.3 (11.1)	59.8 (12.3)	61.1 (10.8)	59.5 (10.8)	60.0 (9.07)

*Sex*						
Female	13,622 (37.3%)	514 (28.9%)	5,524 (37.2%)	232 (28.5%)	173 (37.0%)	4 (16.7%)
Male	22,933 (62.7%)	1,266 (71.1%)	9,329 (62.8%)	582 (71.5%)	295 (63.0%)	20 (83.3%)

*Laterality*						
Left	17,982 (49.2%)	889 (49.9%)	7,302 (49.2%)	429 (52.7%)	248 (53.0%)	12 (50.0%)
Right	18,573 (50.8%)	891 (50.1%)	7,551 (50.8%)	385 (47.3%)	220 (47.0%)	12 (50.0%)

*Race*						
Asian	2,163 (5.9%)	131 (7.4%)	908 (6.1%)	62 (7.6%)	468 (100%)	24 (100%)
Black	3,986 (10.9%)	134 (7.5%)	1,671 (11.3%)	44 (5.4%)	0 (0%)	0 (0%)
Others	720 (2.0%)	27 (1.5%)	299 (2.0%)	12 (1.5%)	0 (0%)	0 (0%)
White	29,686 (81.2%)	1,488 (83.6%)	11,975 (80.6%)	696 (85.5%)	0 (0%)	0 (0%)

*T_stage*						
T1	24,358 (66.6%)	190 (10.7%)	10,281 (69.2%)	84 (10.3%)	393 (84.0%)	8 (33.3%)
T2	3,920 (10.7%)	310 (17.4%)	1,550 (10.4%)	140 (17.2%)	29 (6.2%)	7 (29.2%)
T3	7,580 (20.7%)	1,012 (56.9%)	2,855 (19.2%)	476 (58.5%)	42 (9.0%)	6 (25.0%)
T4	697 (1.9%)	268 (15.1%)	167 (1.1%)	114 (14.0%)	4 (0.9%)	3 (12.5%)

*N_stage*						
N0	34,928 (95.5%)	1,171 (65.8%)	14,437 (97.2%)	557 (68.4%)	452 (96.6%)	18 (75.0%)
N1	1,627 (4.5%)	609 (34.2%)	416 (2.8%)	257 (31.6%)	16 (3.4%)	6 (25.0%)

*Tumor_size*						
Mean (SD)	53.6 (37.4)	102 (48.9)	51.1 (34.4)	101 (55.6)	42.9 (22.0)	68.6 (27.0)

*Histology*						
8120	126 (0.3%)	24 (1.3%)	45 (0.3%)	11 (1.4%)	0 (0%)	0 (0%)
8255	926 (2.5%)	80 (4.5%)	373 (2.5%)	45 (5.5%)	0 (0%)	0 (0%)
8260	4,202 (11.5%)	70 (3.9%)	1,777 (12.0%)	28 (3.4%)	22 (4.7%)	0 (0%)
8310	24,731 (67.7%)	1,146 (64.4%)	10,164 (68.4%)	535 (65.7%)	427 (91.2%)	21 (87.5%)
8312	4,060 (11.1%)	269 (15.1%)	1,544 (10.4%)	113 (13.9%)	16 (3.4%)	3 (12.5%)
8317	1,550 (4.2%)	14 (0.8%)	654 (4.4%)	6 (0.7%)	0 (0%)	0 (0%)
Others	960 (2.6%)	177 (9.9%)	296 (2.0%)	76 (9.3%)	3 (0.6%)	0 (0%)

*Grade*						
I	4,111 (11.2%)	42 (2.4%)	1,724 (11.6%)	19 (2.3%)	69 (14.7%)	0 (0%)
II	18,545 (50.7%)	330 (18.5%)	7,799 (52.5%)	146 (17.9%)	266 (56.8%)	5 (20.8%)
III	10,779 (29.5%)	747 (42.0%)	4,260 (28.7%)	324 (39.8%)	109 (23.3%)	12 (50.0%)
IV	3,120 (8.5%)	661 (37.1%)	1,070 (7.2%)	325 (39.9%)	24 (5.1%)	7 (29.2%)

LM, lung metastasis; 8120, transitional cell carcinoma; 8255, adenocarcinoma with mixed subtypes; 8260, papillary adenocarcinoma; 8310, clear cell adenocarcinoma; 8312, renal cell carcinoma; 8317, chromophobe renal carcinoma.

**Table 3:** Univariate and multivariate logistic regression analysis.

Variables	Univariate analysis		Multivariate analysis	
	OR	*P*	OR	*P*
**Age**	**1.012**	**<0.001**	**1.007**	**0.003**

*Laterality*				
Left	Reference	Reference	Reference	Reference
Right	0.968	0.515		

*Sex*				
Female	Reference	Reference	Reference	Reference
Male	**1.49**	**<0.001**	**1.184**	**0.004**

*Race*				
Asian	Reference	Reference	Reference	Reference
Black	**0.539**	**<0.001**	**0.613**	**<0.001**
Other	**0.604**	**0.019**	**0.604**	**0.037**
White	**0.818**	**0.033**	0.821	0.063

*T_stage*				
T1	Reference	Reference	Reference	Reference
T2	**10.922**	**<0.001**	**4.591**	**<0.001**
T3	**19.599**	**<0.001**	**5.885**	**<0.001**
T4	**79.462**	**<0.001**	**11.172**	**<0.001**

*N_stage*				
N0	Reference	Reference	Reference	Reference
N1	**17.245**	**<0.001**	**4.415**	**<0.001**
**Tumor_size**	**1.024**	**<0.001**	**1.009**	**<0.001**

*Histology*				
8120	Reference	Reference	Reference	Reference
8255	**0.401**	**<0.001**	1.083	0.782
8260	**0.071**	**<0.001**	**0.462**	**0.007**
8310	**0.206**	**<0.001**	1.217	0.451
8312	**0.301**	**<0.001**	1.419	0.192
8317	**0.038**	**<0.001**	**0.196**	**<0.001**
Other	0.96	0.868	1.497	0.142

*Grade*				
I	Reference	Reference	Reference	Reference
II	**1.755**	**<0.001**	1.235	0.214
III	**7.213**	**<0.001**	**2.264**	**<0.001**
IV	**26.042**	**<0.001**	**3.438**	**<0.001**

The bold values indicate that the *p* value is less than 0.05.

**Table 4:** Predictive performance of algorithms in internal and external test cohorts.

Models	Internal test							External test						
	AUC	Accuracy	Sensitivity	Specificity	Precision	Recall	*F*1 score	AUC	Accuracy	Sensitivity	Specificity	Precision	Recall	*F*1 score
LR	0.903	0.787	0.873	0.783	0.180	0.873	0.299	0.896	0.866	0.75	0.872	0.231	0.75	0.353
RF	0.791	0.885	0.661	0.897	0.260	0.661	0.373	0.703	0.876	0.5	0.895	0.197	0.5	0.283
SVM	0.61	0.938	0.326	0.972	0.389	0.326	0.355	0.674	0.919	0.167	0.957	0.167	0.167	0.167
XGB	0.913	0.812	0.873	0.809	0.200	0.873	0.325	0.904	0.872	0.75	0.878	0.240	0.75	0.364
DT	0.820	0.831	0.763	0.835	0.202	0.763	0.319	0.690	0.896	0.458	0.919	0.224	0.458	0.301
ANN	0.906	0.792	0.875	0.787	0.184	0.875	0.304	0.891	0.843	0.792	0.846	0.209	0.792	0.331

LR: logistic regression, RF: random forest, SVM: supporting vector machine, XGB: extreme gradient boosting, DT: decision tree, and ANN: artificial neural network.

## Data Availability

The relevant data and code associated with the current manuscript have been uploaded to the following website https://github.com/qq731936287/kun.git.

## Supplementary Materials

Supplementary Materials Supplementary file 1: the detailed hyperparameters in various machine learning models.
