# Supplementary material for: Development and External Validation of Machine Learning-Based Models for Predicting Lung Metastasis in Kidney Cancer: A Large Population-Based Study
Source: Int J Clin Pract. 2023 Jun 20;2023:8001899. doi: 10.1155/2023/8001899 (PMC10299882; doi:10.1155/2023/8001899)
Supplement: Supplementary Materials — Supplementary file 1: the detailed hyperparameters in various machine learning models. [file 8001899.f1.docx]

Extreme gradient boosting: {parameters (

mtry(range = c(2, 8)),

min_n(range = c(5, 20)),

tree_depth(range = c(1, 3)),

learn_rate(range = c(-3, -1)),

loss_reduction(range = c(-3, 0)),

sample_prop(range = c(0.8, 1))

)}

Support vector machines: {parameters (cost (range = c (-5, 5)),

rbf_sigma(range = c(-4, -1))) }

Decision trees: {parameters (tree_depth(range = c(3, 7)),

min_n(range = c(5, 10)),

cost_complexity(range = c(-6, -1)))}

Random forest: {parameters (

mtry(range = c(2, 5)),

trees(range = c(60, 140)),

min_n(range = c(50, 100))

)}

Artificial neural network: {parameters (hidden_units(range = c(15, 24)),

penalty (range = c(-3, 0)),

epochs (range = c(50, 150)))}
